# Supplementary material for: Directed exciton transport highways in organic semiconductors
Source: Nat Commun. 2023 Sep 12;14:5599. doi: 10.1038/s41467-023-41044-9 (PMC10497625; doi:10.1038/s41467-023-41044-9)
Supplement: Supplementary file 1 — Supplemental Information [file 41467_2023_41044_MOESM1_ESM.pdf]

Supporting Information for

Directed Exciton Transport Highways in Organic Semiconductors

Kai Müller[1,2], Dr. Karl S. Schellhammer[1,3], Dr. Nico Gräßler[3,4], Bipasha Debnath[4], Dr. Fupin Liu[4], Dr. Yulia Krupskaya[4], Prof. Karl Leo[3], Prof. Martin Knupfer[4], Prof. Frank Ortmann[1,5,*]

[1] Center for Advancing Electronics Dresden, Technische Universität Dresden, 01062 Dresden, Germany

[2] Institut für Theoretische Physik, Technische Universität Dresden, 01062 Dresden, Germany

[3] Dresden Integrated Center for Applied Physics and Photonic Materials (IAPP) and Institute for Applied Physics, Technische Universität Dresden, 01062 Dresden, Germany

[4] Leibniz Institute for Solid State and Materials Research Dresden, Helmholtzstr. 20, 01069 Dresden, Germany

[5] TUM School of Natural Sciences, Department of Chemistry, Technische Universität München, Lichtenbergstr. 4, 85748 Garching b. München

* frank.ortmann@tum.de

Content

Supplementary Figures 3

Supplementary Tables 4

Supplementary Methods 12

Material parameterization using density functional theory-based methods 12

Excited States and Interactions 13

Additional Information to the Simulation of TD and other Excitons 16

Supplementary References 18

Supplementary Figures

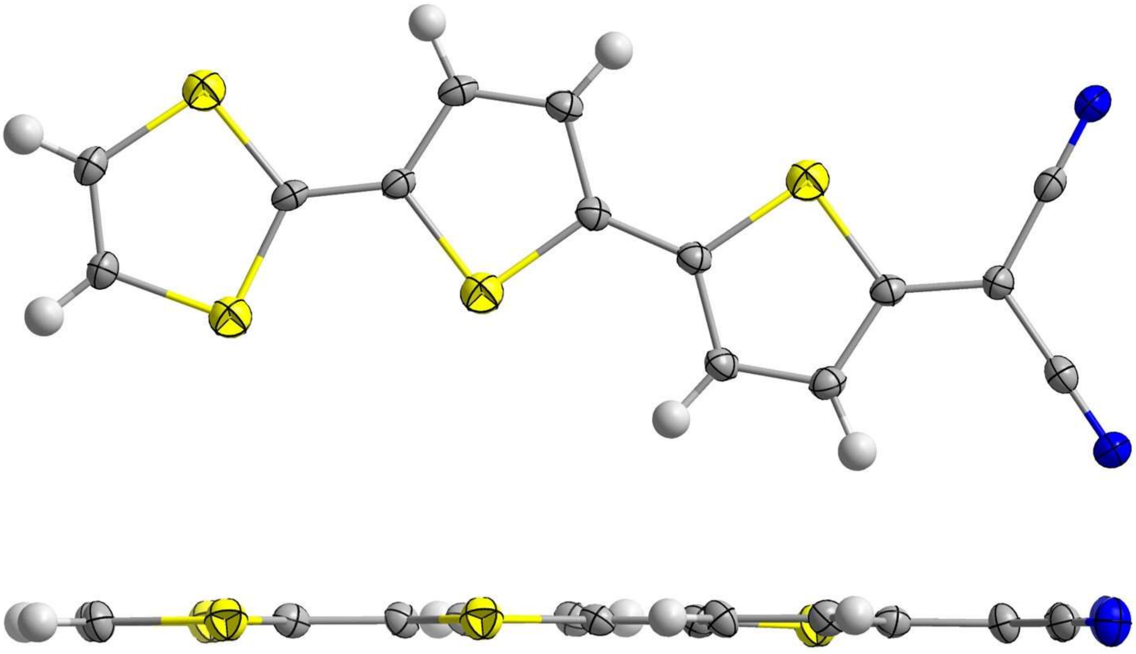

Supplementary Figure 1. Molecular structure drawing at 50% ellipsoid probability. Front view (up) and side view (down) are shown. Color code: gray for carbon, blue for nitrogen, yellow for sulfur and white for hydrogen.

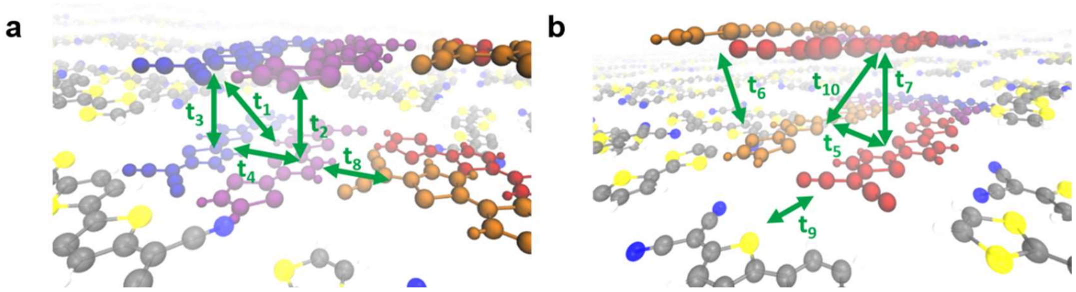

Supplementary Figure 2. Illustration of the nearest neighbour transfer integrals from Supplementary Table 5. (a) Side view illustrating the transfer integrals t_1, t_2, t_3, t_4 and t_8; (b) Alternative view illustrating the remaining transfer integrals t_5, t_6, t_7, t_9 and t_{10}.

Supplementary Tables

Supplementary Table 1. Crystal data of QM1.

**Crystal**	**QM1**
**Formula**	$C_{14}H_6N_2S_4$
**Formula weight**	330.45
**Color, habit**	Red, block
**Crystal system**	triclinic
**Space group**	$P\bar{1}$
***a*, Å**	3.8100(8)
***b*, Å**	16.770(3)
***c*, Å**	20.930(4)
***α*, deg**	94.98(3)
***β*, deg**	91.39(3)
***γ*, deg**	95.85(3)
**Volume, $Å^3$**	1324.6(5)
Z	4
***T*, K**	100
**Radiation (λ, Å)**	Synchrotron Radiation (0.77977)
**Unique data (R_{int})**	3781 (0.0414)
**Parameters**	361
**Restraints**	0
**Observed data ($I > 2\sigma(I)$)**	3462
**R_1^a (observed data)**	0.0357
**wR_2^b (all data)**	0.0990
**CCDC NO.**	2172187

[a]For data with $I > 2\sigma(I)$, $R_1 = \frac{\sum||F_o|-|F_c||}{\sum|F_o|}$. [b]For all data, $wR_2 = \sqrt{\frac{\sum[w(F_o^2-F_c^2)^2]}{\sum[w(F_o^2)^2]}}$.

Supplementary Table 2. Properties of the lowest excited singlet states of a single QM1 molecule in gas phase calculated with TD-DFT (M06-2X/cc-pVTZ) and red-shifted by a solid-state shift. For higher-energy states (state 12 and above), an increased mixing of molecular orbitals leads to a larger number and reduced individual weight of the indicated transitions.

State number	Excitation energy [eV]	Oscillator strength	Dominant state transition
1	1.50	1.33	HOMO −> LUMO
2	2.75	0.00	HOMO-1 −> LUMO
3	2.78	0.00	HOMO −> LUMO+2
4	3.09	0.00	HOMO −> LUMO+1
5	3.40	0.02	HOMO −> LUMO+3
6	3.52	0.08	HOMO-2 −> LUMO
7	3.67	0.02	HOMO-3 −> LUMO
8	3.78	0.00	HOMO −> LUMO+4
9	3.89	0.00	HOMO −> LUMO+6
10	4.33	0.02	HOMO-4 −> LUMO
11	4.44	0.14	HOMO −> LUMO+5
12	4.51	0.01	HOMO-1 −> LUMO+2,<br>HOMO −> LUMO+8
13	4.69	0.00	HOMO −> LUMO+8,<br>HOMO −> LUMO+12
14	4.84	0.00	HOMO-1 −> LUMO+1
15	4.89	0.00	HOMO −> LUMO+12<br>HOMO-5 −> LUMO
16	4.97	0.00	HOMO-5 −> LUMO

Supplementary Table 3. List of the 10 largest interaction energies $J(\mathbf{n}, j = 1, \mathbf{n}', j')$ between two excitons in real space, where exciton 1 is located on molecule 1 in the unit cell (see Figure 2) and exciton 2 at molecule j′. The exciton state number is taken from the TDDFT results listed in Supplementary Table 2. If exciton 1 is located on molecule 2 in the unit cell, the interaction values are identical up to the order of 1meV and are therefore not listed separately here.

Unit cell position j' of exciton 2	State number exciton 1	State number exciton 2	Relative unit cell distance in lattice vectors $\mathbf{n}' - \mathbf{n}$	Interaction strength [meV]
1	1	1	(1,0,0)	133
2	1	1	(0,0,0)	-103
2	1	1	(1,0,0)	-94
2	1	1	(-1,0,0)	56
1	4	4	(1,0,0)	54
1	1	1	(2,0,0)	47
2	1	1	(2,0,0)	-42
2	1	1	(0,1,0)	39
3	1	1	(1,1,1)	-33
1	11	11	(1,0,0)	31

Supplementary Table 4. List of the 10 largest interaction energies $J(\mathbf{n}, j = 3, \mathbf{n}', j')$ between two excitons in real space, where exciton 1 is located on molecule 3 in the unit cell (see Figure 2) and exciton 2 at molecule j′. The exciton state number is taken from the TDDFT results listed in Supplementary Table 2. If exciton 1 is located on molecule 4 in the unit cell, the interaction values are identical up to the order of 1meV and are therefore not listed separately here.

Unit cell position j' of exciton 2	State number exciton 1	State number exciton 2	Relative unit cell distance in lattice vectors $\mathbf{n}' - \mathbf{n}$	Interaction strength [meV]
3	1	1	(1,0,0)	128
4	1	1	(-1,0,0)	-97
4	1	1	(0,0,0)	-78
4	1	1	(-2,0,0)	-51
3	4	4	(1,0,0)	44
3	1	1	(2,0,0)	41
4	1	1	(1,0,0)	-36
4	1	1	(0,-1,0)	36
1	1	1	(-1,-1,-1)	-33
1	1	1	(-2,-1,0)	-30

Supplementary Table 5. List of the 10 largest hole transfer integrals, where the indexes correspond to those shown in Figure 2. Note that transfer integrals that are related by hermiticity are listed only once.

Index of the hole orbital in the unit cell	Index of the hole orbital in the unit cell	Relative unit cell distance in lattice vectors	Hole transfer Integral t_h [meV]
1	2	(0,0,0)	51
1	1	(1,0,0)	51
2	2	(1,0,0)	50
1	2	(1,0,0)	-44
3	4	(-1,0,0)	-26
4	4	(1,0,0)	22
3	3	(1,0,0)	22
1	4	(0,1,1)	-18
3	2	(-1,0,0)	-18
3	4	(0,0,0)	18

Supplementary Table 6. List of the 10 largest electron transfer integrals, where indexes correspond to those shown in Figure 2. Note that transfer integrals that are related by hermiticity are listed only once.

Index of the electron orbital in the unit cell	Index of the electron orbital in the unit cell	Relative unit cell distance in lattice vectors	Electron transfer Integral t_e [meV]
1	2	(1,0,0)	33
1	2	(0,0,0)	-29
1	1	(1,0,0)	27
2	2	(1,0,0)	27
3	4	(-1,0,0)	24
3	4	(0,0,0)	-15
1	4	(-1,1,1)	-9
2	3	(2,0,0)	-9
3	2	(-1,0,0)	7
3	3	(1,0,0)	-7

Supplementary Table 7. Optical band gap and exciton data of the lowest excited singlet states of reference organic semiconductors

Material	Optical gap (eV)	Exciton band width (meV)	Technique	Reference
Naphthalene	3.9	24	OAS	https://doi.org/10.1080/15421407508083209
	3.9	18.7	OAS	https://doi.org/10.1080/15421406608083285
Anthracene	3.12	31	OAS	https://doi.org/10.1080/15421407508083209
	3.14	31	OAS	https://doi.org/10.1007/BF00529393
	3.2	37	OAS + PL	https://iopscience.iop.org/article/10.7567/JJAPS.34S1.212
Phenanthrene	3.54	7	OAS	https://doi.org/10.1080/15421406608083285
1,2-Benzanthracene	3.3	18.5	OAS	https://link.springer.com/article/10.1007/BF00529393
1,2;5,6-Dibenzanthracene	3.4	12.3	OAS	https://link.springer.com/article/10.1007/BF00529393
Tetracene	2.45	74	OAS	https://link.springer.com/article/10.1007/BF00529393
	2.3	85	OAS	https://aip.scitation.org/doi/full/10.1063/1.3590871
	2.3	70	EELS	https://iopscience.iop.org/article/10.120

				9/0295-5075/112/37004/meta
Pentacene, thin film phase	1.9	118	OAS	https://link.springer.com/article/10.1007/BF00529393
	1.87	114	OAS	https://doi.org/10.1039/C8CP06384B
Pentacene, single crystal phase	1.8	123	OAS	https://pubs.acs.org/doi/full/10.1021/acs.jpcc.5b07163
	1.835	142	OAS	https://doi.org/10.1039/C8CP06384B
	1.8	110	EELS	https://aip.scitation.org/doi/full/10.1063/1.4723812
Hexacene	1.46	117	OAS	https://doi.org/10.1515/zna-1974-0227
	1.41	180	Reflectivity	https://doi.org/10.1021/jz501693g
Chrysene	3.3	≤ 20	EELS	https://aip.scitation.org/doi/full/10.1063/1.4753999
1,2-Benzpentacene	1.97	111	OAS	https://link.springer.com/article/10.1007/BF00529393
1,2:8,9-Dibenzpentacene	2.12	99	OAS	https://link.springer.com/article/10.1007/BF00529393
	2.05	70	EELS	https://doi.org/10.1021/acsomega.2c01987
Picene	3.2	≤ 10	EELS	https://journals.aps.org/prb/abstract/10.1

				103/PhysRevB.83.165436
Coronene, high T phase	2.87	60.7	OAS	https://www.sciencedirect.com/science/article/pii/S0022231304004120
Coronene, low T phase	2.9	45.8	OAS	https://doi.org/10.1117/12.236181
Pyrene	3.29	40.9	OAS	https://doi.org/10.1117/12.236181
β-Perylene	2.56	119	OAS	https://doi.org/10.1143/JPSJ.51.1871
Rubrene	2.2	≤ 10	OAS	https://doi.org/10.1103/PhysRevB.75.245416
Quaterthiophene (4T)	2.6	1210	OAS + PL	https://doi.org/10.1063/1.481804
Quinquethiophene (5T)	2.4	1299	OAS + PL	https://doi.org/10.1063/1.481804
Sexihiophene (6T)	2.27	1320	OAS + PL	https://doi.org/10.1063/1.481804
p-Distyrylbenzene (3PV)	3	1000	OAS + PL	https://doi.org/10.1021/acs.chemrev.7b00581
p-Quaterphenyl (4P)	3.5	244	EELS	https://doi.org/10.1063/5.0058657
dinaphtho[2,3-b:2,3-f]thieno[3,2-b]thiophene (DNTT)	2.75	595		https://doi.org/10.1021/acs.jpcc.5b12686
C_{10}-DNTT	2.65	697	OAS + PL	https://doi.org/10.1021/acs.jpcc.5b12686

3,4,9,10-perylene tetracarboxylic dianhydride (PTCDA)	1.9	240	PL	https://doi.org/10.1103/PhysRevB.81.155208
	1.9	200	EELS	https://doi.org/10.1016/S0301-0104(99)00082-8
N,N-bis(phenyl) perylene diimide (N-phenyl PDI)	2.1	50	OAS + PL	https://doi.org/10.1021/acs.jpcc.9b04429
N,N-bis(n-octyl)-2,5,8,11-tetraphenyl perylene diimide (tetraphenyl PDI)	2.1	≤ 12	OAS + PL	https://doi.org/10.1021/acs.jpcc.9b04429
syn-dimethylanthradithiophenes (syn-DMADT)	2.31	100	OAS	unpublished
anti-dimethylanthradithiophenes (anti-DMADT)	2.35	76	OAS	unpublished
7,8,15,16-tetraazaterrylene (TAT)	2.04	16	OAS + PL	https://doi.org/10.1021/jp509011u
N,N-bis(3-pentyl)-perylene-3,4,9,10-bis(dicarboximide) (B2)	1.98	152	OAS	https://doi.org/10.1021/acs.jpclett.7b00283
diF (triethylsilyl)ethynyl -anthradithiophene (diF TES-ADT)	2.32	45	OAS + PL	https://doi.org/10.1063/5.0026072
diF tertbutyldimethylsilylethynyl - anthradithiophene (diF TBDMS-ADT)	2.19	90	OAS + PL	https://doi.org/10.1063/5.0026072
3,3X-disulfopropyl-X Ž .5,5 -dichloro-9-ethyl thiacarbocyanine (THIATS)	2	330	OAS + PL	https://doi.org/10.1016/S0009-2614(97)01290-6
Open-ring photomerocyanine spirooxazines Py-SO)	2.1	130	IXS	https://doi.org/10.1103/PhysRevLett.98.036404
β-nickel phthalocyanine (NiPc)	1.85	90	EELS	https://doi.org/10.1021/ac

				s.jpcc.1c02654
β-copper phthalocyanine (CuPc)	1.76	20	EELS	https://doi.org/10.1021/acs.jpcc.1c02654
β-zinc phthalocyanine (ZnPc)	1.73	≤ 10	EELS	https://doi.org/10.1021/acs.jpcc.1c02654
Para-nitroaniline (PNA)	2.8	302	OAS	https://doi.org/10.1016/0009-2614(78)85150-1
C_{60}	1.8	≤ 12	EELS	https://doi.org/10.1016/S0167-5729(00)00012-1

Supplementary Methods

Material parameterization using density functional theory-based methods

Electronic and optical material properties were simulated using density functional theory (DFT) based methods. The experimental crystal structure information was used as initial geometry for a relaxation of the position of hydrogen atoms within the fixed unit cell. This calculation is performed using the PBE functional[1] for the description of the electron-ion interaction within the VASP software package[2]. Dispersion force corrections are essential for van-der-Waals bonded systems[3] and we include the DFT-D3 van der Waals correction with Becke-Johnson damping.[4]

The structures of the individual molecules within the unit cell were extracted to calculate the static dipole moment by DFT calculations with the GAUSSIAN16 software package[5] in combination with the M06-2X functional and the cc-pVTZ basis set using a Hirshfeld population analysis.[6–8] The same level of theory was used in combination for time-dependent DFT calculations (TD-DFT) [9–12] to extract the properties of the singlet excited states, i.e. the excitation energies (gas phase), the oscillator strengths, the transition densities, and the relaxation energy for the first excited state. The transition density and CHELPG transition

charges[13] were extracted from the TD-DFT calculations by using Multiwfn.[14,15] The gas-phase excitation energies were reduced by a solid state shift of 0.6 eV, which takes into account stronger screening effects in the bulk, lowering the excitation energy. It is obtained empirically because this number is not in the focus of our work. To calculate the transfer integrals from the relaxed unit cell, the B3LYP functional and the 6-311G** basis set were applied.[16–20] The method proposed by Kubar *et al.* including the Löwdin transformation was used to extract the absolute values of the transfer integrals.[21] Their sign was obtained from an evaluation of the orientation of the orbitals within the corresponding dimer and monomer calculations.

Excited States and Interactions

We consider a set of molecular exciton (ME) and charge transfer (CT) excitons in our model. The molecular excitons, which are transitions between the highest occupied molecular orbital (HOMO) and the lowest unoccupied molecular orbital (LUMO) in the first part of our work, are described in the spirit of the Frenkel exciton model using the most general notation

$$H_{\mathrm{ME}} = E_{\mathrm{ME}} \sum_{\mathbf{N}} |\mathbf{N},\mathbf{N}\rangle\langle\mathbf{N},\mathbf{N}| + \sum_{\mathbf{M},\mathbf{N}} J_{\mathbf{M},\mathbf{N}} |\mathbf{M},\mathbf{M}\rangle\langle\mathbf{N},\mathbf{N}|, \qquad (1)$$

where the orbital indexes $\mathbf{N}$ and $\mathbf{M}$ run over all the molecules in the crystal and the basis is understood as $|\mathbf{N}_{\mathrm{hole}},\mathbf{M}_{\mathrm{electron}}\rangle$. E_{ME} are the molecular exciton energies in the solid (ME site energies) and $J_{\mathbf{M},\mathbf{N}}$ the ME couplings. The latter are Coulomb mediated terms which can be represented in a multipole expansion, leading, for example, to the approximate transition dipole coupling approach. Here we avoid this approximation for $J_{\mathbf{M},\mathbf{N}}$ by using directly the transition densities (vide infra) from TD-DFT simulations as described in section 2 above.

The translational symmetry allows introducing in equation (1) the lattice notation for the orbital position (center of mass) $\mathbf{N} = \mathbf{n} + \mathbf{r}_j$, indicating the unit cell position by a lattice vector $\mathbf{n}$ and the orbital position $\mathbf{r}_j$ relative to the origin of the unit cell, leading to the Frenkel Hamiltonian in the lattice notation

$$H_{\mathrm{ME}} = E_{\mathrm{ME}} \sum_{\mathbf{n},j} |\mathbf{n}+\mathbf{r}_j,\mathbf{n}+\mathbf{r}_j\rangle\langle\mathbf{n}+\mathbf{r}_j,\mathbf{n}+\mathbf{r}_j|$$

$$+ \sum_{\mathbf{n},j,\mathbf{n}',j'} J(\mathbf{n},j,\mathbf{n}',j') \, |\mathbf{n}+\mathbf{r}_j,\mathbf{n}+\mathbf{r}_j\rangle\langle\mathbf{n}'+\mathbf{r}_{j'},\mathbf{n}'+\mathbf{r}_{j'}|. \qquad (2)$$

Excitonic coupling is described through the Coulomb interaction

$$J(\mathbf{n},j,\mathbf{n}',j') = \sum_{\alpha,\alpha'} \frac{T_{\alpha\mathbf{n}j} T_{\alpha'\mathbf{n}'j'}}{4\pi\varepsilon_0\varepsilon_r|\mathbf{R}|} (1 - \delta_{\mathbf{n},\mathbf{n}'}\,\delta_{j,j'}), \qquad (3)$$

in which $T_{\alpha\mathbf{n}j}$ is the transition density at atom α of molecule $(\mathbf{n},j)$ for the ME states, distance vector $\mathbf{R} = \mathbf{n}' - \mathbf{n} + \mathbf{r}_{j'\alpha'} - \mathbf{r}_{j\alpha}$ between the atoms, and ε_r is set to 2. Note that a simpler

transition-dipole coupling-based interaction approach that could be obtained by a two-fold series expansion of the distance R between of the transition densities and introduction of TD multipoles, has difficulties in convergence for the close molecular contact and is not used here for quantitative results. A more refined modelling could consider additional non-local atomic transition densities which are commonly smaller than the used intramolecular ones, decay exponentially and are not included here. The same holds for their overlaps.

Due to the sizable transfer integrals between molecules, we additionally consider (HOMO-LUMO type) excitations with CT character, i.e. excitations in which the orbitals are located on neighboring molecules. The CT states are described by

$$H_{\mathrm{CT}} = \sum_{\mathbf{n},j,\mathbf{s}\neq 0} E_{\mathrm{CT}}(\mathbf{r}_j, \mathbf{s})|\mathbf{n} + \mathbf{r}_j, \mathbf{n} + \mathbf{r}_j + \mathbf{s}\rangle\langle\mathbf{n} + \mathbf{r}_j, \mathbf{n} + \mathbf{r}_j + \mathbf{s}|. \qquad (4)$$

The CT energies $E_{\mathrm{CT}}(\mathbf{r}_j, \mathbf{s})$ depend only on the relative position of HOMO and LUMO orbitals, which is given by the position vector of the hole ($\mathbf{r}_j$) and the vector pointing from hole to electron ($\mathbf{s}$) in the CT pair. The CT excitons included in our calculation (that is the set $\{\mathbf{s}\}$ over which the above sum runs) are described in Sect. 4 below. In highly symmetric atomic crystals, the CT energy might only depend on the difference vector but in the present case, we need to take the positions of electron and hole into account. $E_{CT}(\mathbf{r}_j, \mathbf{s})$ is therefore described relative to the hole position.

While in absence of any coupling between the excitons, the set of electron-hole pair states (CT and ME states) constitutes the manifold of uncoupled states. The presence of transfer integrals, however, requires the extension of the Hamiltonian by introducing the terms

$$H_{\mathrm{coupl}} = \sum_{\mathbf{n},j,\mathbf{s},\boldsymbol{a}} t_e(\mathbf{n}, j, \mathbf{s}, \boldsymbol{a})|\mathbf{n} + \mathbf{r}_j, \mathbf{n} + \mathbf{r}_j + \mathbf{s}\rangle\langle\mathbf{n} + \mathbf{r}_j, \mathbf{n} + \mathbf{r}_j + \mathbf{s} + \boldsymbol{a}|$$
$$+ \sum_{\mathbf{n},j,\mathbf{s},\boldsymbol{a}} t_h(\mathbf{n}, j, \mathbf{s}, \boldsymbol{a})|\mathbf{n} + \mathbf{r}_j, \mathbf{n} + \mathbf{r}_j + \mathbf{s}\rangle\langle\mathbf{n} + \mathbf{r}_j + \boldsymbol{a}, \mathbf{n} + \mathbf{r}_j + \mathbf{s}|, \qquad (5)$$

where $t_{e(h)}$ are the transfer integrals for electrons (holes) and $\boldsymbol{a}$ represents the vector between the pair of coupled orbitals involved. Note that the transfer integrals induce electronic interactions between ME and CT states on the one hand and between different CT states on the other. Transfer integrals in stacking direction of the molecules in the studied crystal are comparable in size to the ones in the non-stacking directions, such as between the dimers (see Supplementary Figure 2). Besides transfer integrals, another interaction between CT and ME states could occur, namely the Coulomb interaction. However, since CT oscillator strengths are small and CT transition dipoles are usually orders of magnitude lower than $|\mathbf{d}_i|$, Coulomb

coupling is not considered between ME and CT states or between different CT states. This concludes the setup of our Hamiltonian in real space.

An efficient treatment of the Hamiltonian exploits the translational symmetry for the center of mass coordinate. Here, for concreteness of the representation, we use the hole position as the reference position for the exciton. Note that this introduces an *insignificant* asymmetry in the representation of the Hamiltonian between electrons and holes. We transform the Hamiltonian into momentum space ($\mathbf{q}$ in the Brillouin zone) by introducing the notation (N_Ω is the number of unit cells)

$$|\mathbf{q}; \mathbf{r}_j, \mathbf{r}_j + \mathbf{s}\rangle = \frac{1}{\sqrt{N_\Omega}} \sum_{\mathbf{n}} e^{i\mathbf{q}(\mathbf{n}+\mathbf{r}_j)} |\mathbf{n} + \mathbf{r}_j, \mathbf{n} + \mathbf{r}_j + \mathbf{s}\rangle. \quad (6)$$

The inverse transformation to Eq. (6) reads

$$|\mathbf{n} + \mathbf{r}_j, \mathbf{n} + \mathbf{r}_j + \mathbf{s}\rangle = \frac{1}{\sqrt{N_\Omega}} \sum_{\mathbf{q}} e^{-i\mathbf{q}(\mathbf{n}+\mathbf{r}_j)} |\mathbf{q}; \mathbf{r}_j, \mathbf{r}_j + \mathbf{s}\rangle. \quad (7)$$

Applied to the Hamiltonian, we get

$$H_{\mathrm{ME}} = E_{\mathrm{ME}} \sum_{\mathbf{q}} \sum_j |\mathbf{q}; \mathbf{r}_j, \mathbf{r}_j\rangle\langle\mathbf{q}; \mathbf{r}_j, \mathbf{r}_j| + \sum_{\mathbf{q},j,j'} J(\mathbf{q}, j, j') |\mathbf{q}; \mathbf{r}_j, \mathbf{r}_j\rangle\langle\mathbf{q}; \mathbf{r}_{j'}, \mathbf{r}_{j'}|, \quad (8)$$

with

$$J(\mathbf{q}, j, j') = \sum_{\boldsymbol{m}} e^{i\mathbf{q}(\boldsymbol{m}-\mathbf{r}_j+\mathbf{r}_{j'})} J(0, j, \boldsymbol{m}, j'). \quad (9)$$

We continue with the block diagonalization of H_{coupl} in equation (5). Having translational symmetry for the transfer integrals means that they do not depend on the cell index $\mathbf{n}$. i.e. $t_{\mathrm{e(h)}}(\mathbf{n}, j, \mathbf{s}, \boldsymbol{a}) \to t_{\mathrm{e(h)}}(j, \mathbf{s}, \boldsymbol{a})$. We can write the electron coupling part of the Hamiltonian of equation (5)

$$H_{\mathrm{coupl,e}} = \sum_{\mathbf{q},j,s,a} t_e(j, \mathbf{s}, \boldsymbol{a}) |\mathbf{q}; \mathbf{r}_j, \mathbf{r}_j + \mathbf{s}\rangle\langle\mathbf{q}; \mathbf{r}_j, \mathbf{r}_j + \mathbf{s} + \boldsymbol{a}|, \quad (10)$$

which has a block diagonal form in which the diagonalization w.r.t. $\mathbf{q}$ is explicit.
For the electronic coupling between holes we obtain

$$H_{\mathrm{coupl,h}} = \sum_{\mathbf{q},j,j',s,a} t_h(j, \mathbf{s}, -\boldsymbol{a}) e^{-i\mathbf{q}\boldsymbol{a}} \delta_{\{\mathbf{r}_j-\boldsymbol{a}\},\mathbf{r}_{j'}} |\mathbf{q}; \mathbf{r}_j, \mathbf{r}_j + \mathbf{s}\rangle\langle\mathbf{q}; \mathbf{r}_{j'}, \mathbf{r}_{j'} + \mathbf{s} + \boldsymbol{a}|. \quad (11)$$

This resulting hole transfer term is more complicated in its form than the electron transfer term. What is different as compared to electrons is that the hole is effectively shifted into the original unit cell when (through $\boldsymbol{a}$) it was displaced in a neighboring one. This introduces the phase factor $e^{-i\mathbf{q}\boldsymbol{a}}$. The second difference is that the molecular index of the hole might change from $\mathbf{r}_j$ to $\{\mathbf{r}_j - \boldsymbol{a}\}$ in which the curly brackets denote the corresponding reduced vector in the new unit cell. We emphasize that the apparent asymmetry between electrons and holes is not a true physical asymmetry but is a consequence of the particular choice we have to make by

introducing explicitly the distance vector between electron and hole in the pair-state notation. All results are independent of this choice.

Together with the electron transfer integral terms (equation (10)) and the onsite CT part in its block-diagonalized form

$$H_{\mathrm{CT}} = \sum_{\mathbf{q},j,j',\mathbf{s}\neq 0} E_{CT}(\mathbf{r}_j, \mathbf{s})\delta_{jj'} |\mathbf{q}; \mathbf{r}_j, \mathbf{r}_j + \mathbf{s}\rangle\langle \mathbf{q}; \mathbf{r}_{j'}, \mathbf{r}_{j'} + \mathbf{s}| \quad (12)$$

and the Frenkel part (8) we have all matrix elements to set up the excitonic Bloch Hamiltonian in the matrix representation

$$H(\mathbf{q}, j, j', \mathbf{s}, \mathbf{s}') = t_{\mathrm{h}}(j, \mathbf{s}, \mathbf{s} - \mathbf{s}')e^{i\mathbf{q}(\mathbf{s}'-\mathbf{s})}\delta_{\{\mathbf{r}_j - \mathbf{s}'+\mathbf{s}\},\mathbf{r}_{j'}} + t_{\mathrm{e}}(j, \mathbf{s}, \mathbf{s}' - \mathbf{s})\delta_{jj'}$$

$$+E_{\mathrm{CT}}(\mathbf{r}_j, \mathbf{s})(1 - \delta_{\mathbf{s},0})\delta_{\mathbf{s},\mathbf{s}'}\delta_{jj'} + \left(E_{\mathrm{ME}}\delta_{jj'} + J(\mathbf{q}, j, j')\right)\delta_{\mathbf{s}0}\delta_{\mathbf{s},\mathbf{s}'}. \quad (13)$$

For all molecules j, we select the neighbors $\mathbf{s}(j)$, which yields 4x7 exciton states per unit cell, hence a rank 28 of the excitonic Hamiltonian. Note that when matrix elements are assigned, there can be contributions from hole transfer and electron transfer on the same Bloch matrix elements.

The solutions of the eigenvalue equation for the exciton Hamiltonian are given in terms of the coefficients $c_{\nu,\mathbf{q}}(\mathbf{r}_j, \mathbf{s})$ in the chosen representation according to

$$\sum_{\mathbf{s}',j'} H(\mathbf{q}, j, j', \mathbf{s}, \mathbf{s}')c_{\nu,\mathbf{q}}(\mathbf{r}_{j'}, \mathbf{s}') = E_\nu(\mathbf{q})c_{\nu,\mathbf{q}}(\mathbf{r}_j, \mathbf{s}). \quad (14)$$

Additional Information to the Simulation of TD and other Excitons

For a transparent extended FE-CT model and to restrict the number of states in our model to a reasonable size, we only include those CT states that are directly coupled to ME states by sizeable hole or electron transfer integrals. In other words, only CT excitons were included where the electron-hole pair is separated by a vector $\mathbf{s} \neq 0$ in which $\mathbf{s}$ corresponds to a minimum-sized transfer integral (small electron-hole separation in the CT state). In principle, charge-separated (weakly bound or unbound) CT states could also be included, however they are not relevant due to their negligible oscillator strength and are not considered here. Supplementary Figure 2 provides an overview over the important transfer integrals that are taken into account in the simulations. We note that the transfer integrals t_{e} and t_{h} between LUMO and HOMO orbitals, respectively, are calculated in DFT (see above for details) and reach values up to 50 meV. From all transfer integrals we select only those with a magnitude above 11 meV. Taking smaller transfer integrals into account did not change the results significantly but increased the complexity substantially, which justifies our choice.

The energy of the CT states was calculated based on the Coulomb law with screened interaction, the Frenkel exciton energy and the simulation parameter $E_{\text{shift}} = 0.7$ eV, which fixes the energy difference between the Frenkel exciton and the CT exciton separated by the shortest lattice vector $|\boldsymbol{a}_x|$. For a general CT exciton with the charges separated by a larger distance r, we interpolate the energy of the state with a Coulomb law as

$$E_{\text{CT}}(r) = E_{\text{Frenkel}} + E_{\text{shift}} + \frac{e^2}{4\pi\varepsilon_0\varepsilon_r}\left(\frac{1}{|\boldsymbol{a}_x|} - \frac{1}{r}\right). \tag{15}$$

Supplementary References

1. Perdew, J. P., Burke, K. & Ernzerhof, M. Generalized gradient approximation made simple. *Phys. Rev. Lett.* **77**, 3865–3868 (1996).
2. Kresse, G. & Furthmüller, J. Efficient iterative schemes for ab initio total-energy calculations using a plane-wave basis set. *Phys. Rev. B - Condens. Matter Mater. Phys.* **54**, 11169–11186 (1996).
3. Ortmann, F., Bechstedt, F. & Schmidt, W. G. Semiempirical van der Waals correction to the density functional description of solids and molecular structures. *Phys. Rev. B - Condens. Matter Mater. Phys.* **73**, 205101 (2006).
4. Grimme, S., Ehrlich, S. & Goerigk, L. Effect of the damping function in dispersion corrected density functional theory. *J. Comput. Chem.* **32**, 1456–1465 (2011).
5. Frisch, M. J. *et al.* G16_C01. Gaussian 16, Revision C.01, Gaussian, Inc., Wallin at (2016).
6. Hirshfeld, F. L. Bonded-atom fragments for describing molecular charge densities. *Theor. Chim. acta 1977 442* **44**, 129–138 (1977).
7. Ritchie, J. P. Electron Density Distribution Analysis for Nitromethane, Nitromethide, and Nitramide. *J. Am. Chem. Soc.* **107**, 1829–1837 (1985).
8. Ritchie, J. P. & Bachrach, S. M. Some methods and applications of electron density distribution analysis. *J. Comput. Chem.* **8**, 499–509 (1987).
9. Zhao, Y. & Truhlar, D. G. The M06 suite of density functionals for main group thermochemistry, thermochemical kinetics, noncovalent interactions, excited states, and transition elements: two new functionals and systematic testing of four M06-class functionals and 12 other function. *Theor. Chem. Acc.* **120**, 215–241 (2008).
10. Dunning, T. H. Gaussian basis sets for use in correlated molecular calculations. I. The atoms boron through neon and hydrogen. *J. Chem. Phys.* **90**, 1007–1023 (1989).
11. Kendall, R. A., Dunning, T. H. & Harrison, R. J. Electron affinities of the first-row atoms revisited. Systematic basis sets and wave functions. *J. Chem. Phys.* **96**, 6796–6806 (1992).
12. Stratmann, R. E., Scuseria, G. E. & Frisch, M. J. An efficient implementation of time-dependent density-functional theory for the calculation of excitation energies of large molecules. *J. Chem. Phys.* **109**, 8218–8224 (1998).
13. Breneman, C. M. & Wiberg, K. B. Determining atom-centered monopoles from molecular electrostatic potentials. The need for high sampling density in formamide

conformational analysis. *J. Comput. Chem.* **11**, 361–373 (1990).

14. Madjet, M. E., Abdurahman, A. & Renger, T. Intermolecular coulomb couplings from ab initio electrostatic potentials: Application to optical transitions of strongly coupled pigments in photosynthetic antennae and reaction centers. *J. Phys. Chem. B* **110**, 17268–17281 (2006).
15. Lu, T. & Chen, F. Multiwfn: A multifunctional wavefunction analyzer. *J. Comput. Chem.* **33**, 580–592 (2012).
16. Gaussian 16, Revision B.01, Frisch, M. J. et al. , Gaussian, Inc., W. C. Gaussian 16. (2016).
17. Becke, A. D. A new mixing of Hartree–Fock and local density-functional theories. *J. Chem. Phys.* **98**, 1372–1377 (1993).
18. Becke, A. D. Density-functional exchange-energy approximation with correct asymptotic behavior. *Phys. Rev. A* **38**, 3098–3100 (1988).
19. Becke, A. D. Density-functional thermochemistry. III. The role of exact exchange. *J. Chem. Phys.* **98**, 5648–5652 (1993).
20. Krishnan, R., Binkley, J. S., Seeger, R. & Pople, J. A. Self-consistent molecular orbital methods. XX. A basis set for correlated wave functions. *J. Chem. Phys.* **72**, 650–654 (1980).
21. Kubař, T., Woiczikowski, P. B., Cuniberti, G. & Elstner, M. Efficient Calculation of Charge-Transfer Matrix Elements for Hole Transfer in DNA. *J. Phys. Chem. B* **112**, 7937–7947 (2008).
